# Delivering Digital Health Solutions that Patients Need: A Call to Action

**DOI:** 10.1007/s43441-023-00592-4

**Published:** 2023-12-11

**Authors:** Veronica Popa, Jan Geissler, Rebecca Vermeulen, Elizabeth Priest, Katherine Capperella, Gözde Susuzlu, Sharon F. Terry, Nicholas Brooke

**Affiliations:** 1 EURORDIS, Bucharest, Romania; 2 Patvocates, Hohenbrunn, Germany; 3 Roche, Redwood City, CA USA; 4 The Synergist.Org and PFMD, Barcelona, Spain; 5 Johnson & Johnson, Horsham, PA USA; 6 Data Saves Lives, Brussels, Belgium; 7 Genetic Alliance, Damascus, MD USA; 8 The Synergist.Org and PFMD, Brussels, Belgium

**Keywords:** Patient engagement, Digital health, Patient involvement, Digital data governance, Patient-centric design

## Abstract

Digital health solutions have the potential to complement traditional healthcare approaches and deliver improved health outcomes, but there are system-wide challenges that need to be addressed. These include fragmentation of the digital health landscape, regulatory processes that lack the agility to accommodate the fast pace of digital health advances, and inadequate transparency around data sharing and data governance. All of these challenges have led to mistrust, limited understanding and sharing of best practices, a lack of digital education and awareness, and insufficient patient and public engagement and involvement. In this paper, we argue that for digital health solutions to fulfil their potential, there needs to be a significant increase in early, meaningful, and sustained engagement with the people they intend to benefit. The uptake as well as the impact of digital solutions created in partnership with patients for patients are greater and more relevant to the communities they address.

## Introduction

The value of patient and public/citizen involvement and engagement in healthcare is widely accepted and has been demonstrated in research and medicines development [1–7]. The benefits of involvement to patients and other health stakeholders have also been described in research priority settings, clinical trial design, regulatory processes, and health technology assessments [6, 8]. Consequently, patient engagement (PE) is increasingly being incorporated in drug development [9], with the aim of delivering more effective healthcare solutions for improved treatment outcomes. In this context, the term “patient” encompasses not only those with a condition, but all those impacted by the condition, including carers and relatives; hence, the term is not restrictive or intended to reduce the person to a patient [10].

Digital health solutions have been identified as an essential strategy to strengthen health systems [11, 12] and improve health outcomes, but there are challenges that first need to be addressed. These include fragmentation of the digital health landscape [13, 14], regulatory barriers [15, 16], lack of transparency and data sharing (to support further research and improve research efficiency) [17–20], limited identification and sharing of best practice [21, 22], insufficient digital health education and awareness [23, 24], and lack of patient involvement [25] (Table 1). This paper focuses on the value of embedding PE within digital health approaches to address these challenges. In this context, PE means working with patients as partners to identify and articulate needs, design and co-create solutions, and help define data governance requirements.

**Table 1.** Key Challenges and Barriers to Delivering Digital Health Solutions that Meet Patients’ Needs

Theme	Challenges and Barriers
Fragmented approach to digital health	• Rapid emergence of private organizations developing and marketing their own digital health solutions for commercial purposes without integration, coherence, or focus on the patient perspective • Fragmented landscape of siloed initiatives that fail to enhance patient experience and acceptance, exacerbated by the large number of stakeholders and complexity of the digital environment • Initiatives deliver only a fraction of their potential digital health value and fail to positively impact patient outcomes because they are not designed with patients • Across private and public sectors, the need to quickly deliver convenience has been prioritized over production of solutions with patients that maximize health outcomes for patients • Disjointed digital health solutions were created in response to urgent needs during the COVID-19 pandemic • Lack of integration of digital services into healthcare from the patient perspective
Regulatory landscape	• Regulatory process developed for conventional health solutions that lack the agility and flexibility to accommodate the digital health development approach (e.g., “constant beta” approach that allows rapid adaptation in line with changes in the industry landscape)
Transparency and data sharing	• Lack of transparency around data sharing and data governance, which stifles collaboration and drives rivalry and mistrust, leading to insular solutions and subsequently lack of adoption • Lack of clarity around health and non-health data
Identification and sharing of best practice	• Lack of process or platforms for identifying and sharing best practice and for evaluating whether digital solutions are truly tailored to patients’ needs and preferences • Low awareness of whether patients have the necessary resources to adopt/use digital solutions optimally, or see value in the solutions
Education and awareness	• Lack of education for patients and patient representatives to gain a wider understanding of the digital environment and how to engage with it • Lack of education for healthcare providers to support patients with identifying and accessing relevant digital health solutions and services
Patient involvement	• Low awareness amongst developers of why, when, and how to engage with patients, and of the value of return on investment • Lack of prioritization of patient contribution to the design, development, and adoption of digital health solutions or in needs assessment, service design, and data governance • Limited awareness of the difference between user-centric design and patient-centric design • Patient involvement generally limited to beta testing of an already designed digital solution • Lack of patient-validated digital endpoints for patient outcomes

## Value of PE in Digital Health

Meaningful PE improves understanding of patients’ needs and preferences, ensuring that digital health solutions are aligned with priorities of patients, that there is an appropriate balance between benefit and cost, and that ethical boundaries are respected. Patients, patient advocates, and patient organizations can play an important role in digital health; they can help increase impact and improve outcomes through the design, development, acceptance, and adoption of digital solutions. A patient-directed approach is also essential to establishing transparency and good governance concerning digital data [26, 27]. Patient organizations have the capability to become key partners in data management by acting as data custodians or stewards, supporting data collection and data sharing [28, 29]. From a development perspective, including patients early in decision making can also prevent incorrect priorities, mitigate blind spots, and deter costly redesigns.

Where comparable, digital health technologies are relatively unfettered by the regulatory and safety considerations that govern development and approval of conventional medical interventions and devices. There is also no formal assessment or standardized validation of digital health solutions to evaluate whether they deliver the “benefit” they purport to deliver [21, 30]. Arguably, given this somewhat unconstrained environment, PE throughout the digital health development process becomes even more important to ensure that the end solution has real value for the intended users (patients) and can be integrated into their lives. Co-production with patients is essential to deliver solutions that improve health outcomes and emphasize social and societal accountability.

## Barriers to PE in Digital Health

A systematic review of global patient and public involvement in digital health solutions concluded that involvement is seen as “valuable and essential in digital health innovation, but rarely practised” [25], reflecting that barriers to PE in digital health are numerous and multifactorial. Digital technologies are advancing quickly with increasing applications in digital health [21]. A key consequence of this rapidly evolving marketplace is fragmentation of the landscape [21], with many stakeholders and a complex digital environment hindering patient involvement. In this fast-paced, disjointed environment it may not seem as practical to embed PE in the development of digital health solutions compared to the relatively longer timeline for medicines development [31]. In addition, due to the competitive nature of the environment, patients are often not involved or consulted in solutions that are created, minimizing the value and overall impact the potential technology could bring. Paradoxically, fragmentation makes PE increasingly important as only patients can identify which solutions are truly beneficial. In practical terms, there is a need to address fragmentation by “mapping” the digital health landscape, its technologies, applications, related issues, and stakeholders to better structure the discussion around PE in digital health.

The fast pace of digital health further highlights that PE should be planned and implemented as early as possible to maximize opportunities for patient-centric development. Patient-centric design should be translated into digital health, using (and developing if needed) the appropriate tools and language to communicate with patients and consumers. There also needs to be a collaborative effort, including insight from patients, experienced patient advocates and patient organization representatives, to look at digital health from a patient pathway perspective and identify key value creation points for patients (Table 2).

**Table 2.** Key Next Steps to Trigger the Early and Consistent Inclusion of PE in Digital Development

• Patients should be involved as valued partners in decision-making from the start and throughout the development process, not just as passive beneficiaries of the end solution • Sufficient budgeting for PE should be included at the onset of development • Patient experts, patient advocates, and patient organizations should be part of the co-design of data governance models and digital data management plans • Digital solutions and collaborative initiatives should be transparent in how and when patients have been involved • Patients should also be consulted regarding the business sustainability models	

Other barriers to PE in digital health include lack of trust around digital health data management [32–35], exacerbated by a lack of transparency in quality and stewardship and little leadership or standardization outside of General Data Protection Regulation. While investment in digital health continues to grow [36, 37], funds are rarely set aside for PE as part of the development process, and PE is not often prioritized or embedded in the workflow. While limited digital literacy from the patient perspective and technology company competence in engaging patients can also be barriers to PE, not including patients in digital health technology design and development leads to a lack of adoption and insular implementation since patients do not see the value of, or even mistrust, the resulting digital health solutions and services. Importantly, the patient community and patient organizations may need to acquire certain capabilities in order to fully engage with and co-create digital health solutions. These needs should be identified and addressed at project initiation and further developed through direct participation in digital projects. Opening communication channels is key to facilitating insightful and potentially transformative discussions between patients and developers.

Digital health conversations generally have a deeply rooted mercantile aspect: the focus is more often on technological solutions (such as wearables, telehealth, or health information technology) and convenience. This is understandable as organizations are operating in the world they know where interoperability, safe data sharing, and patient return on investment may not be well understood or part of the organizational culture. Institutional digital health stakeholders should include patient organizations in the co-design of data governance models and digital data management plans, leveraging governance best-practices to develop operational models which support trusted patient information sharing, optimizing data contribution to research and ultimately business sustainability. Patient organizations, thought leaders, advocates, and experts can play an important role in the digital and data protection formation of patient communities, adhering to Findability, Accessibility, Interoperability, and Reusability (FAIR) guiding principles [38] and establishing processes that encourage data sharing for patient benefit.

## Need for Multistakeholder Collaboration in Digital Health

Multistakeholder collaboration could help to address barriers by collaborating to advance solutions that are truly meaningful for patients. The value of co-production in healthcare is increasingly acknowledged [39]. This could be translated to digital health as a co-creation model where all parties benefit. The World Health Organization has stated, “Digital health should be an integral part of health priorities and benefit people in a way that is ethical, safe, secure, reliable, equitable, and sustainable. It should be developed with principles of transparency, accessibility, scalability, replicability, interoperability, privacy, security, and confidentiality” [12]. Multistakeholder collaboration and meaningful PE in needs assessment, design, development, and adoption of digital health solutions will be essential to reach these ambitious but attainable goals. There is little documented evaluation on how patients have been involved in digital health studies [14], but lessons learned from medicines development [40] demonstrate that patients should be involved early and often if digital health solutions are to meet genuine patient needs and improve health outcomes. While there may be existing multi-stakeholder collaborations for PE in digital health, such as Patient Focused Medicines Development (PFMD), these can be difficult to identify due to lack of transparency of how and when patients have been involved. As such, digital solutions and collaborative initiatives should be transparent in this regard.

## Delivering Digital Health Solutions that Improve Health Outcomes: A Call to Action

A patient-centric, patient-engaged approach is required if digital health solutions are to address patients’ unmet needs and deliver improved health outcomes. In the complex, fast-paced, fragmented digital environment, multistakeholder partnerships are essential to delivering digitally enabled, improved health outcomes. Digital technologies and health solutions from technology, pharmaceutical, and device industries need to be integrated to provide holistic interventions for patients, rather than standalone solutions that are siloed outside existing health and social care services. The collaboration should incorporate meaningful and consistent PE where patients are involved as valued partners in decision-making from the start and throughout the development process, not just as passive beneficiaries of the end solution. This commitment should be made clear through sufficient budgeting for PE at the onset of development. As explained by Papoutsi and colleagues [41], “a shift is needed from co-designing with technology users to co-designing with patients as service users” in digital health.

Patient organizations, patient communities, and non-governmental organizations could serve as integrators between the rapidly evolving digital landscape and the slower regulatory and health technology assessment process, e.g., by prioritizing digital health solutions and potentially speeding up regulatory activity. Learnings from PE in medicines development demonstrate that lay people and participants can be valuable partners in research [42–44]. This highlights that there is also an opportunity for patient communities to have a proactive role in the design of digital health solutions that would deliver meaningful outcomes that matter most to them. The role of the patient community and how to engage with patients (as both the primary source of data and end users of digital health solutions) needs to be better understood and established to unlock the potential of digital and data.

## Conclusion

Multistakeholder collaboration with patients as partners is paramount. It will help deliver high-value, accessible, trusted digital health solutions that reflect patients’ needs and preferences, particularly regarding data safety and privacy; it will also help drive large-scale acceptance and adoption.

## References

[1] Baumann LA, Reinhold AK, Brütt AL (2022). Public and patient involvement in health policy decision-making on the health system level—a scoping review. Health Policy.

[2] Geissler J, Ryll B, di Priolo SL, Uhlenhopp M (2017). Improving patient involvement in medicines research and development: a practical roadmap. Ther Innov Regul Sci.

[3] Manafo E, Petermann L, Mason-Lai P, Vandall-Walker V (2018). Patient engagement in Canada: a scoping review of the ‘how’ and ‘what’ of patient engagement in health research. Health Res Policy Syst.

[4] Levitan B, Getz K, Eisenstein EL (2018). Assessing the financial value of patient engagement: a quantitative approach from CTTI’s patient groups and clinical trials project. Ther Innov Regul Sci.

[5] Forsythe LP, Carman KL, Szydlowski V (2019). Patient engagement in research: early findings from the Patient-Centered Outcomes Research Institute. Health Aff.

[6] Vat LE, Finlay T, Jan Schuitmaker-Warnaar T (2020). Evaluating the “return on patient engagement initiatives” in medicines research and development: a literature review. Health Expect.

[7] Diaby V, Ali AA, Montero AJ (2019). Value assessment frameworks in the United States: a call for patient engagement. Pharmacoecon Open.

[8] Murphy A, Bere N, Vamvakas S (2022). The added value of patient engagement in early dialogue at EMA: scientific advice as a case study. Front Med (Lausanne).

[9] Zvonareva O, Craveț C, Richards DP (2022). Practices of patient engagement in drug development: a systematic scoping review. Res Involv Engagem.

[10] Lalanda M, Gracia-Peligero E, Delgado-Marroquín MT (2017). They are people first, then patients. AMA J Ethics.

[11] Labrique A, Vasudevan L, Mehl G (2018). Digital health and health systems of the future. Glob Health Sci Pract.

[12] World Health Organization 2021. Global strategy on digital health 2020–2025. Available from: https://www.who.int/docs/default-source/documents/gs4dhdaa2a9f352b0445bafbc79ca799dce4d.pdf.

[13] Iyamu I, Gómez-Ramírez O, Xu AX (2022). Challenges in the development of digital public health interventions and mapped solutions: findings from a scoping review. Digit Health.

[14] Van Velthoven MH, Cordon C (2019). Sustainable adoption of digital health innovations: perspectives from a stakeholder workshop. J Med Internet Res.

[15] Lim SY, Anderson EG. ‘Institutional barriers against innovation diffusion: from the perspective of digital health startups’ 2016 49th Hawaii International Conference on System Sciences (HICSS) Koloa, HI, USA, 2016 pp. 3328–3337.

[16] Mureyi D (2022). Overcoming institutionalised barriers to digital health systems: an autoethnographic case study of the judicialization of a digital health tool. BMC Med Inform Decis Mak.

[17] Grundy Q, Chiu K, Held F (2019). Data sharing practices of medicines related apps and the mobile ecosystem: traffic, content, and network analysis. BMJ.

[18] Savage L, Gaynor M, Adler-Milstein J (2018). Digital health data and information sharing: a new frontier for health care competition. Antitrust LJ.

[19] Watts G (2019). Data sharing: keeping patients on board. Lancet Digit Health.

[20] Schwalbe N, Wahl B, Song J, Lehtimaki S (2020). Data sharing and global public health: defining what we mean by data. Front Digit Health.

[21] Mathews SC, McShea MJ, Hanley CL (2019). Digital health: a path to validation. NPJ Digit Med.

[22] Solomon DH, Rudin RS (2020). Digital health technologies: opportunities and challenges in rheumatology. Nat Rev Rheum.

[23] Brown TM, Bewick M (2022). Digital health education: the need for a digitally ready workforce. Arch Dis Child Educ Pract Ed.

[24] van Kessel R, Wong BLH, Clemens T (2022). Digital health literacy as a super determinant of health: more than simply the sum of its parts. Int Interv.

[25] Baines R, Bradwell H, Edwards K (2022). Meaningful patient and public involvement in digital health innovation, implementation and evaluation: a systematic review. Health Expect.

[26] Morey T, Forbath T, Schoop A (2015). Customer data: designing for transparency and trust. Harv Bus Rev.

[27] Smith RJ, Grande D, Merchant RM (2016). Transforming scientific inquiry: tapping into digital data by building a culture of transparency and consent. Acad Med.

[28] van Lin N, Paliouras G, Vroom E (2021). How patient organizations can drive FAIR data efforts to facilitate research and health care: a report of the Virtual Second International Meeting on Duchenne Data Sharing, March 3, 2021. J Neuromuscul Dis.

[29] de Freitas C, Amorim M, Machado H (2021). Public and patient involvement in health data governance (DATAGov): protocol of a people-centred, mixed-methods study on data use and sharing for rare diseases care and research. BMJ Open.

[30] Soobiah C, Cooper M, Kishimoto V (2020). Identifying optimal frameworks to implement or evaluate digital health interventions: a scoping review protocol. BMJ Open.

[31] Brown DG, Wobst HJ, Kapoor A (2021). Clinical development times for innovative drugs. Nat Rev Drug Discov.

[32] Adjekum A, Blasimme A, Vayena E (2018). Elements of trust in digital health systems: scoping review. J Med Internet Res.

[33] Leeming G, Ainsworth J, Clifton DA (2019). Blockchain in health care: hype, trust, and digital health. Lancet.

[34] Ruotsalainen P, Blobel B (2020). Health information systems in the digital health ecosystem—problems and solutions for ethics, trust and privacy. Int J Environ Res Public Health.

[35] Augustin C, Holeman I, Salomon E (2021). Pathways to increasing trust in public health data: an exploratory analysis of quality issues and potential remediation for data collected using the community health toolkit. Chance.

[36] Pifer R. The shifting digital health investment landscape in 2022. Available from: https://www.healthcaredive.com/news/digital-health-VC-investment-landscape-2022/617063/.

[37] Joseph S. What bubble? Digital health funding year in review 2021. Available from: https://www.forbes.com/sites/sethjoseph/2022/01/11/what-bubble-digital-health-funding-year-in-review-2021/.

[38] Wilkinson MD, Dumontier M, Aalbersberg IJ (2016). The FAIR guiding principles for scientific data management and stewardship. Sci Data.

[39] Elwyn G, Nelson E, Hager A (2020). Coproduction: when users define quality. BMJ Qual Saf.

[40] Hoos A, Anderson J, Boutin M (2015). Partnering with patients in the development and lifecycle of medicines: a call for action. Ther Innov Regul Sci.

[41] Papoutsi C, Wherton J, Shaw S (2021). Putting the social back into sociotechnical: case studies of co-design in digital health. J Am Med Inform Assoc.

[42] Terry SF (2017). The study is open: participants are now recruiting investigators. Sci Transl Med.

[43] Browne T, Swoboda A, Ephraim PL (2020). Engaging patients and family members to design and implement patient-centered kidney disease research. Res Involv Engagem.

[44] Tong A, Scholes-Robertson N, Hawley C (2022). Patient-centred clinical trial design. Nat Rev Nephrol.

